# The Potential of Enamel Matrix Derivative in Countering Bisphosphonate-Induced Effects in Osteoblasts

**DOI:** 10.3390/life14091088

**Published:** 2024-08-29

**Authors:** Minah Kim, Minji Choi, Yong-Dae Kwon, Joo-Young Ohe, Junho Jung

**Affiliations:** 1 Department of Oral & Maxillofacial Surgery, Kyung Hee University College of Dentistry, Kyung Hee University Medical Center, Seoul 02447, Republic of Korea; 2 Division of Oral & Maxillofacial Surgery, Department of Dentistry, Saint Vincent’s Hospital, The Catholic University of Korea, Suwon 16247, Republic of Korea; 3 Department of Dentistry, Graduate School, Kyung Hee University, Seoul 02447, Republic of Korea; 4 Clinical Research Institute, Kyung Hee University Medical Center, Seoul 02447, Republic of Korea

**Keywords:** enamel matrix derivative, enamel matrix protein, bisphosphonate, medication-related osteonecrosis of the jaw

## Abstract

The suppressive effect of bisphosphonates (BPs) on bone metabolism is considered to be a major cause of medication-related osteonecrosis of the jaw (MRONJ). Enamel matrix derivative (EMD) stimulates and activates growth factors, leading to the regeneration of periodontal tissues. In this study, we aimed to explore the potential of EMD in reversing the detrimental effects of BPs on human fetal osteoblasts (hFOBs) and osteosarcoma-derived immature osteoblasts (MG63s) by assessing cell viability, apoptosis, migration, gene expression, and protein synthesis. While the suppressive effect of zoledronate (Zol) on cell viability and migration was observed, the addition of EMD significantly mitigated this effect and enhanced cell viability and migration. Furthermore, an increased apoptosis rate induced by Zol was decreased with the addition of EMD. The decreased gene expression of alkaline phosphatase (ALP), osteocalcin (OC), and the receptor activator of nuclear factors kappa-B ligand (RANKL) caused by BP treatment was reversed by the co-addition of EMD to hFOB cells. This trend was also observed for ALP and bone sialoprotein (BSP) levels in MG63 cells. Furthermore, suppressed protein levels of OC, macrophage colony-stimulating factor (M-CSF), BSP, and type 1 collagen (COL1) were recovered following the addition of EMD. This finding suggests that EMD could mitigate the effects of BPs, resulting in the recovery of cell survival, migration, and gene and protein expression. However, the behavior of the osteoblasts was not fully restored, and further studies are necessary to confirm their effects at the cellular level and to assess their clinical usefulness in vivo for the prevention and treatment of MRONJ.

## 1. Introduction

Bisphosphonates (BPs) are pyrophosphate-derived synthetic drugs that are commonly used as anti-resorptive agents for patients with osteoporosis, Paget’s disease, osteogenesis imperfecta, and multiple myeloma, as well as for the prevention of skeletal-related events and the treatment of bone metastases associated with malignancies such as breast and prostate cancers [1,2,3,4,5,6]. BPs influence bone metabolism by inhibiting bone resorption mediated by osteoclasts, thereby decreasing bone remodeling [7]. Additionally, these drugs can affect impact various cell types, including osteoblasts, fibroblasts, and endothelial cells [8], leading to reduced cell proliferation and alterations in the gene and protein expression of various cytokines [9].

Medication-related osteonecrosis of the jaw (MRONJ), first recognized in the early 2000s, is now a well-known complication in patients taking anti-resorptive medications. Although the exact accurate pathogenesis of MRONJ is unclear [10,11], impaired bone remodeling caused by the suppression of osteoclasts has been suggested as a primary mechanism [12]. In addition to the anti-osteoclastic effects of BPs [7], their role in suppressing osteoblasts’ proliferation, differentiation, and activity is thought to contribute to the development of MRONJ by inhibiting bone formation [8,13,14,15,16]. The inhibition of alkaline phosphatase (ALP) expression, along with the suppression of bone-related proteins, disrupts osteoblast differentiation, especially in the early stages of osteoblast lineage, such as in osteoprogenitor cells [15]. Several studies have shown that BPs not only inhibit ALP and osteocalcin (OC) expression, but also suppress early osteoblast differentiation by hindering the expression of other extracellular matrix proteins, specifically type 1 collagen (COL1), a marker of early osteoblast differentiation [9,17,18]. Moreover, given that the macrophage colony-stimulating factor (M-CSF) and the receptor activator of nuclear factors κB ligand (RANKL) that are secreted by osteoblasts regulate osteoclastogenesis, the role of osteoblasts cannot be overlooked [19,20].

Several pharmacological strategies are being considered to prevent the development of MRONJ and improve treatment outcomes, including the use of antibiotics, antiseptic mouth rinses, hyperbaric oxygen therapy, teriparatide as a parathyroid hormone analog, and various experimental pharmacological substances [10,21,22,23]. However, these strategies have yet to be established as effective measures for the prevention and treatment of MRONJ.

Enamel matrix derivative (EMD) is composed of enamel proteins such as amelogenin, enamelin, ameloblastin, amelotin, and apin, along with various proteinases extracted from porcine fetal teeth [24], and it can promote the periodontal regeneration of alveolar bone, the periodontal ligament, and cementum [25]. Several studies have demonstrated that EMD stimulates bone formation by enhancing osteoblastic activity, local growth factor expression, protein secretion, and mineral deposition [26,27,28,29]. Therefore, we posit that the stimulatory effect of EMDs on osteoblasts counteracts the reduction in osteoblast function and differentiation caused by BPs. However, studies investigating the effects of EMD on osteoblasts under the influence of BPs and the potential of EMD in treating and preventing MRONJ remain scarce.

In this study, we aimed to investigate the effect of EMD on osteoblasts under the influence of BPs in vitro. Our evaluation focused on several key parameters, including cell viability, apoptosis, migration, and gene and protein expression. Through this comprehensive analysis, we aimed to elucidate the potential of EMD in mitigating the adverse effects of BPs on osteoblast function, thereby providing valuable insights into therapeutic strategies for the prevention and treatment of MRONJ in patients undergoing BP therapy.

## 2. Materials and Methods

### 2.1. Cell Cultures and Treatments

Human fetal osteoblast (hFOB) and osteosarcoma-derived immature osteoblast (MG63) cell lines were obtained from the American Type Culture Collection (Rockville, MD, USA). The hFOB cells were cultured in a 1:1 mixture of Ham’s F12 medium and Dulbecco’s modified Eagle’s medium (DMEM/F12; Gibco, Grand Island, NY, USA) without phenol red. The culture medium was supplemented with 2.5 mM L-glutamine, 0.3 mg/mL G418, penicillin (100 U/mL), streptomycin (100 µg/mL), and 10% fetal bovine serum (FBS; Gibco). MG63 cells were cultured in DMEM supplemented with penicillin (100 U/mL), streptomycin (100 µg/mL), and 10% FBS. Both cell lines were maintained as monolayers in plastic culture plates at 33 °C for hFOB and 37 °C for MG63 in a humidified atmosphere containing 5% CO_2_.

**Figure 1:**
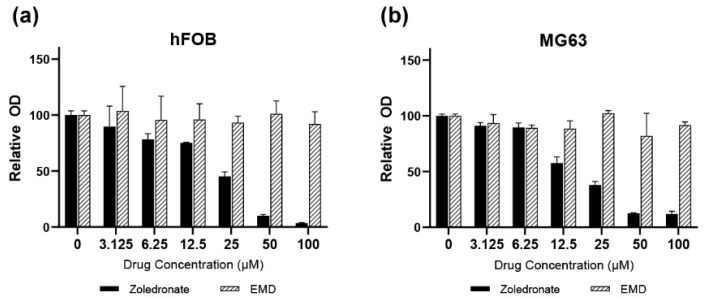
(**a**) hFOB (**b**) MG63 cells. Assessment of hFOB and MG63 cell viability over 72 h using various concentrations of zoledronate and Emdogain (30 μg/mL). EMD, Emdogain.

For the experiments, zoledronic acid/zoledronate (Zol) as a BP and EMD in a liquid formulation (Enamel matrix proteins in acetic acid at a concentration of 30 mg/mL; Straumann AG, Basel, Switzerland) were prepared and added to the wells in the culture plates with working concentrations of 25 μM and 30 μg/mL, respectively. The Zol concentration was determined based on the preliminary experiments conducted in this study (Figure 1). Since Zol decreased cell viability and demonstrated toxicity in a dose-dependent manner, an inhibitory concentration of 50% (EC_50_) as compared to the untreated control cells was used for analysis; thus, a Zol concentration of 25 μM was used for further experiments.

After the cells were seeded onto appropriate plates and incubated for 24 h, they were supplemented with Zol and/or EMD based on the group assignment. The exposure period varied depending on the analytical method used. In the group treated with EMD alone, the cells were not exposed to Zol.

Four experimental groups were established:

Group I (control), cells without Zol and EMD;
Group II (Zol), cells treated with Zol only;
Group III (Zol + EMD), cells treated with Zol and EMD;
Group IV (EMD), cells treated with EMD only.

hFOB and MG63 cells were evaluated for viability, apoptosis, migration, and gene and protein expression. All experiments were performed in triplicate to ensure reproducibility and accuracy.

### 2.2. Cell Viability

Cell viability was assessed using the 3-(4,5-Dimethylthiazol-2-yl)-2,5-diphenyltetrazolium bromide assay (MTT; Sigma-Aldrich, St. Louis, MO, USA), which is a colorimetric method based on the metabolic activity of cells. Cells were seeded in 96-well plates at a density of 2 × 10^5^ cells/cm^2^ and incubated for 24 h. Subsequently, Zol (25 μM) and/or EMD (30 μg/mL) were added to the wells based on the defined experimental groups. After 72 h of incubation, a stock solution of 5 mg/mL MTT dye was prepared in Dulbecco’s phosphate-buffered saline. Subsequently, 20 μL of the MTT dye was added to each well, followed by a 4 h incubation period. After removing the supernatant, 100 μL dimethyl sulfoxide (DMSO; Gibco) was added to solubilize the formed formazan crystals. The plate was then incubated for an additional 15 min. The resulting color was analyzed by measuring the absorbance at 570 nm (A570) using a microplate reader (Bio-Rad Laboratories, Hercules, CA, USA). Cell viability was quantified as a value relative to the control.

### 2.3. Cell Apoptosis Analysis

After 72 h of incubation with Zol and/or EMD, apoptosis was measured by staining the cells with annexin V fluorescein isothiocyanate (FITC; Sigma-Aldrich) and propidium iodide (PI; Sigma-Aldrich). The cells were suspended in binding buffer at a density of 1 × 10^6^ cells/mL and stained with 5 μL of annexin V FITC and 5 μL PI. The cell suspension was then incubated in the dark at room temperature for 15 min. The stained cells were analyzed using a FACS CONTOIITM flow cytometer (BD Biosciences, Franklin Lakes, NJ, USA).

Different cell subpopulations were distinguished based on the following criteria:

Q1: necrotic cells (annexin V-FITC-negative/PI-positive)
Q2: late apoptotic cells (annexin V-FITC-positive/PI-positive)
Q3: viable cells (annexin V-FITC-negative/PI-negative)
Q4: early apoptotic cells (annexin V-FITC-positive/PI-negative)

The apoptosis rate was determined by the sum of the percentages of cells in Q2 and Q4.

### 2.4. Cell Migration

#### 2.4.1. Scratch Wound Healing Assay

hFOB cells were initially seeded in 6-well plates at a density of 3 × 10^5^ per well in DMEM/F12 supplemented with 10% FBS. After 12 h of seeding, the cells were exposed to Zol (25 μM) and/or EMD (30 μg/mL) for 48 h. A scratch was then made in the cell monolayer; the closing of the scratch wound was observed at 24 and 48 h and the size of the gap was calculated using ImageJ software (version 1.54g).

#### 2.4.2. Boyden Chamber Assay

The migration assay was conducted in an AP48-modified Boyden chamber (NeuroProbe, Cabin John, MD, USA). Briefly, the migration chamber consisted of an upper and lower compartment with a porous membrane in between them. The upper compartment was free of chemoattractants, whereas the lower compartment was filled with 0.1% bovine serum albumin (Sigma-Aldrich) as a chemoattractant. Cells were seeded in DMEM/F12 supplemented with 10% FBS at a density of 3 × 10^5^ per well into the upper compartment of the migration assay setup. They were then exposed to Zol (25 μM) and/or EMD (30 μg/mL) and allowed to migrate through a porous membrane to the lower chamber.

### 2.5. Quantitative Reverse Transcription Polymerase Chain Reaction (qRT-PCR) Analysis

qRT-PCR was used to analyze the expression of genes encoding osteogenic and osteoclastogenesis-stimulating markers. Cells were seeded in 60 cm^2^ culture flasks and incubated with Zol (25 μM) and/or EMD (30 μg/mL) for 72 h. Total RNA was extracted using the TRI reagent (Molecular Research Center, Cincinnati, OH, USA) and quantified using a Nanodrop spectrophotometer (ThermoFisher Scientific, Waltham, MA, USA). Primer sequences for genes encoding alkaline phosphatase (ALP), osteocalcin (OC), type 1 collagen (COL1), bone sialoprotein (BSP), macrophage colony-stimulating factor (M-CSF), receptor activator of nuclear factors κB ligand (RANKL), and glyceraldehyde 3-phosphate dehydrogenase (GAPDH) are listed in Table 1.

For cDNA synthesis, reverse transcription was performed with TOPscript^TM^ RT DryMIX (Enxymonics, Daejeon, Republic of Korea). PowerUp SYBR Green Master Mix (ThermoFisher Scientific) was used for PCR reactions. The 2^−ΔΔCT^ method was used to determine RNA expression levels [30], and the expression levels were normalized against GAPDH mRNA levels.

### 2.6. Western Blotting

hFOB cells were incubated for 72 h with Zol and/or EMD, then harvested and lysed in cold lysis buffer (Sigma) for 30 min. The lysates were centrifuged at 4 °C, and the supernatant was collected. Protein content was quantified using a bicinchoninic acid protein assay kit (Pierce, Rockford, IL, USA). Thirty micrograms of protein from each sample was separated using 6–20% sodium dodecyl sulfate-polyacrylamide gel electrophoresis (SDS-PAGE) and transferred to a polyvinylidene difluoride (PVDF) membrane via wet electroblotting. The membranes were blocked with a solution containing 5% dried skimmed milk powder in Tris-buffered saline with Tween^®^ 20 buffer (Bio-Rad Laboratories). They were then incubated overnight at 4 °C with the primary antibodies targeting ALP, BSP, M-CSF, RANKL (Cat. No. ab83259, ab52128, ab9693, and ab9957, respectively; Abcam, Cambridge, UK), OCN, COL1A2, and β-actin (Cat. No. sc-365797, sc-166865, and sc-47778, respectively; Santa Cruz Biotechnology, Dallas, TX, USA). The membranes were then incubated with horseradish peroxidase-conjugated secondary antibodies (Bio-Rad Laboratories). The immunoreactive proteins were determined using an enhanced chemiluminescence detection kit (Amersham Pharmacia Biotech, Amersham, United Kingdom).

### 2.7. Statistical Analysis

One-way analysis of variance (ANOVA), followed by the Tukey’s post-hoc test, was used for normally distributed data. For non-normally distributed data, the Kruskal–Wallis test and Mann–Whitney U test with Bonferroni corrections were applied. Statistical significance was set at *p* < 0.05. All statistical analyses were performed using SPSS software (version 26.0; IBM Corp., Armonk, NY, USA).

## 3. Results

### 3.1. Cell Viability

**Figure 2:**
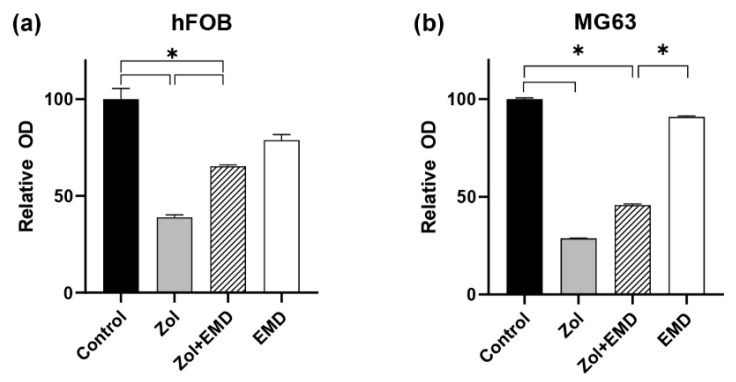
Assessment of cell viability according to the combination of zoledronate and Emdogain. (**a**) hFOB (**b**) MG63 cells. Asterisks (*) indicate statistical significance compared to the control, and the error bars represent standard deviations. Zol, zoledronate; EMD, Emdogain.

A dose-dependent suppressive effect of Zol on cell viability was observed in both hFOB and MG63 cells after 72 h (Figure 1). Cell viability decreased to <50% when the drug concentration reached 25 μM. In hFOB cells treated with Zol, cell viability significantly decreased to 44% compared to the control (*p* < 0.001; Figure 2). However, the addition of EMD significantly mitigated this effect by increasing cell viability (*p* = 0.011), although it remained significantly lower than that of the control (*p* = 0.023). A similar trend was observed in MG63 cells, in which Zol treatment led to a significant decrease in cell viability. However, although the addition of EMD improved cell viability compared to that of the Zol-treated group, this improvement was not statistically significant (*p* = 0.091). In both cell types, supplementation with EMD reversed the negative effects of Zol on cell viability.

### 3.2. Cell Apoptosis

**Figure 3:**
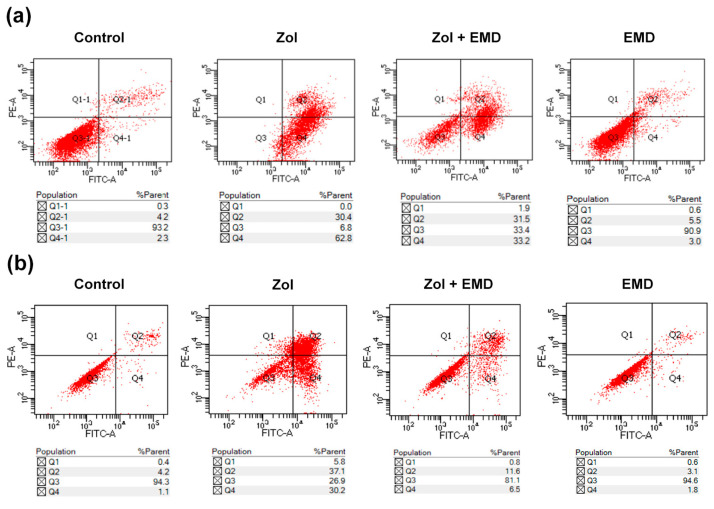
Subpopulation analysis of stained cells with Annexin V-FITC/PI. (**a**) hFOB (**b**) MG63 cells. Necrotic, late apoptotic, viable, and early apoptotic cells appeared in the top left quadrant (Q1), top right quadrant (Q2), bottom left quadrant (Q3) and bottom right quadrant (Q4), respectively. The apoptotic rate is determined as the percentage of cells in Q2 + Q4.

Treatment with Zol markedly increased the apoptosis rate of hFOB cells to 93.2% from 6.5% in the control group. However, in the group treated with both Zol and EMD, the apoptosis rate decreased to 64.7%. In MG63 cells, the Zol-treated group exhibited an increased apoptosis rate of 67.3% compared to 5.3% in the control group. Similar to what was observed in hFOB cells, the apoptosis rate of MG63 cells treated with both Zol and EMD was reduced to 18.1% (Figure 3).

### 3.3. Cell Migration

**Figure 4:**
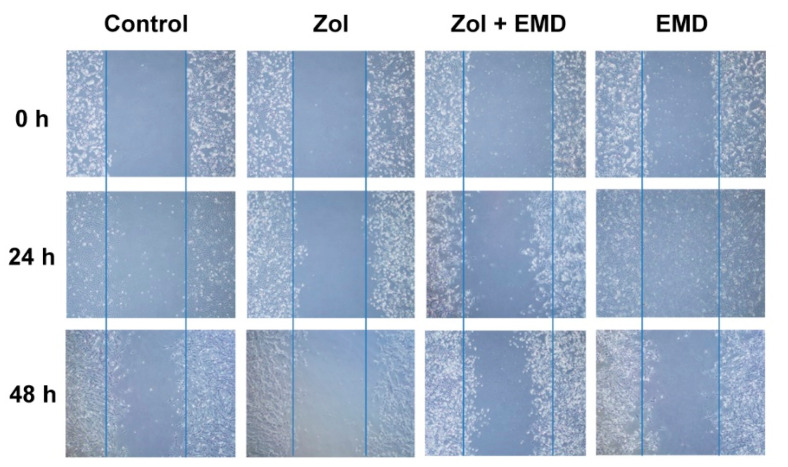
Wound scratch assay of hFOB cells treated with zoledronate and Emdogain over 24 and 48 h. Zol, zoledronate; EMD, Emdogain.

The wound scratch assay results for hFOB cells showed that the wound healing process was notably slowed over time in the Zol-treated group. However, in the group treated with both Zol and EMD, the effect of Zol was not profound, and the width of the scratched wound was similar to that of the control (Figure 4).

**Figure 5:**
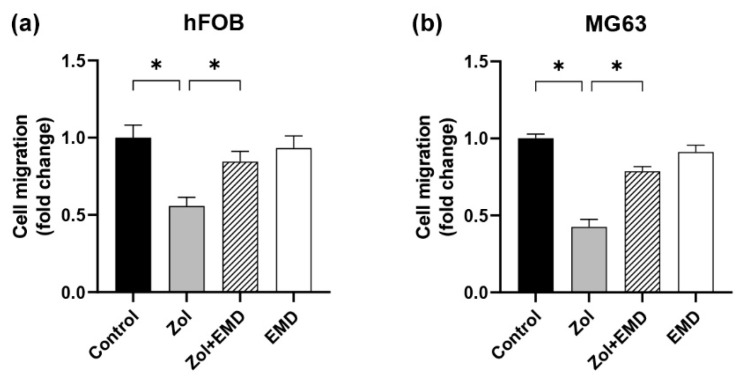
Cell migration assessed using the Boyden chamber assay for (**a**) hFOB and (**b**) MG63 cells. Asterisks (*) indicate statistical significance compared to the control. The error bars represent standard deviations. Zol, zoledronate; EMD, Emdogain.

The fold change in cell migration was measured using the Boyden chamber assay and normalized to that of the control group (set to 1; Figure 5). In hFOB cells, cell migration was significantly decreased in the Zol-treated group compared to that of the control group (*p* < 0.001). However, in the group treated with both Zol and EMD, cell migration significantly increased (*p* = 0.005) and became statistically similar to that of the control. Similarly, in MG63 cells, cell migration was significantly decreased by Zol treatment alone (*p* < 0.001). Conversely, the addition of EMD significantly enhanced cell migration, which was approximately double that of the Zol-treated group (*p* = 0.007).

### 3.4. Gene Expression

**Figure 6:**
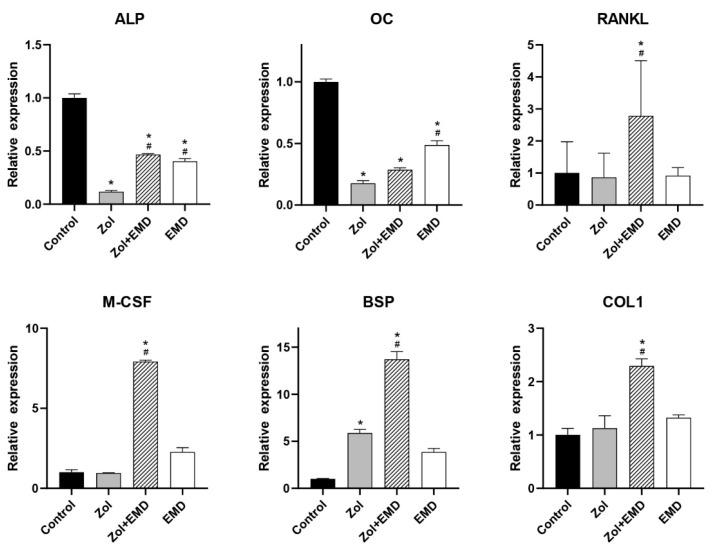
qRT-PCR analysis results for hFOB cells. Gene expression levels were normalized to those of the control. Asterisks (*) indicate statistical significance compared to the control group, while hash signs (#) denote significance compared to the Zol group. The error bars indicate the standard deviation. ALP, alkaline phosphatase; OC, osteocalcin; RANKL, receptor activator of nuclear factor kappa-B ligand; M-CSF, macrophage colony-stimulating factor; BSP, bone sialoprotein; COL1, type 1 collagen; Zol, zoledronate; EMD, Emdogain.

**Figure 7:**
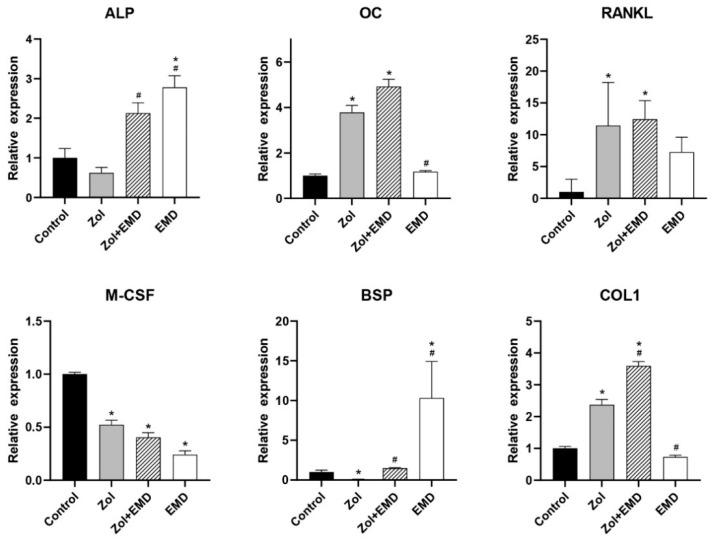
qRT-PCR analysis results for MG63 cells. Gene expression levels were normalized to those of the control. Asterisks (*) indicate statistical significance compared to the control group, while hash signs (#) denote significance compared to the Zol group. The error bars indicate the standard deviation. ALP, alkaline phosphatase; OC, osteocalcin; RANKL, receptor activator of nuclear factor kappa-B ligand; M-CSF, macrophage colony-stimulating factor; BSP, bone sialoprotein; COL1, type 1 collagen; Zol, zoledronate; EMD, Emdogain.

Figure 6 and Figure 7 show the qRT-PCR analysis results for gene expression levels in hFOB and MG63 cells. In hFOB cells, Zol treatment alone significantly reduced the expression of ALP; however, ALP expression levels were significantly upregulated with the co-addition of EMD compared with that of the Zol-treated group (*p* = 0.047). Zol treatment did not reduce M-CSF, BSP, and COL1 expression levels, whereas the co-addition of EMD significantly increased their expression. In MG63 cells, treatment with Zol resulted in decreased expression of ALP, M-CSF, and BSP, whereas co-treatment with EMD and Zol led to a significant increase in the expression of ALP and BSP. Additionally, while the expression of OC, RANKL, and COL1 was significantly increased compared to that of the control, these markers also showed a significant increase when treated with Zol alone.

### 3.5. Protein Expression

**Figure 8:**
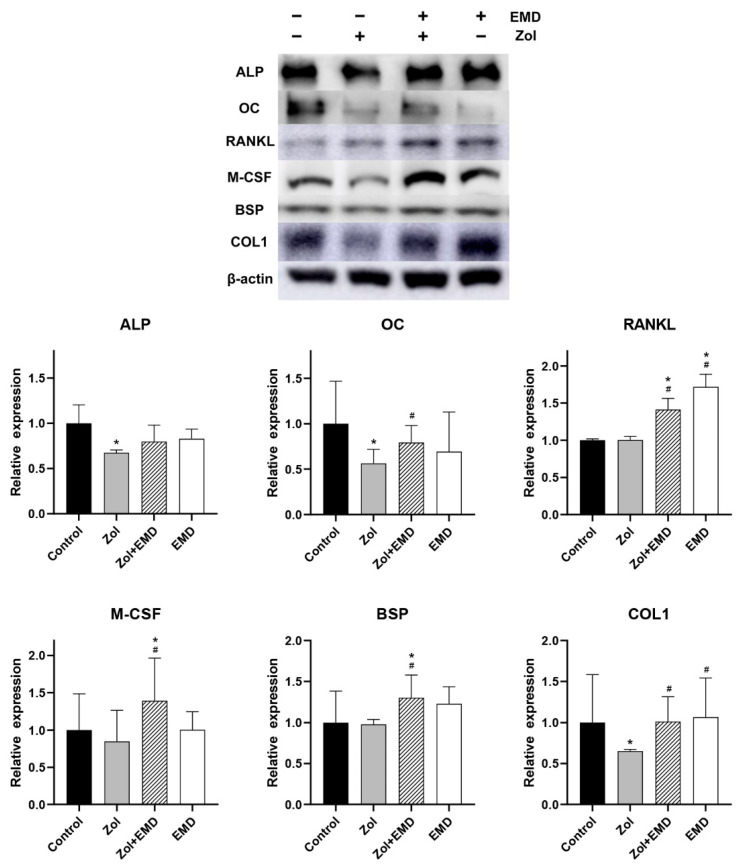
Western blot analysis results of protein expression in hFOB cells. Levels were normalized to those of the control. Asterisks (*) indicate statistical significance compared to the control group, while hash signs (#) denote significance compared to the Zol group. The error bars indicate the standard deviation. ALP, alkaline phosphatase; OC, osteocalcin; RANKL, receptor activator of nuclear factor kappa-B ligand; M-CSF, macrophage colony-stimulating factor; BSP, bone sialoprotein; COL1, type 1 collagen; Zol, zoledronate; EMD, Emdogain.

Compared to that of the control, the expression levels of ALP, OC, M-CSF, and COL1 were decreased in the Zol-treated group, with significant decreases observed for ALP, OC, and COL1 (Figure 8). Although the addition of EMD to Zol treatment resulted in an increased expression of all proteins, only the results for OC, RANKL, M-CSF, BSP, and COL1 were statistically significant compared to those of the Zol-only group. Decreases in RANKL and BSP levels were not notable after Zol treatment; however, their levels increased when EMD and Zol were administered together, as compared to those of the control group.

## 4. Discussion

Although the exact pathophysiology of MRONJ remains unclear, it is now considered a multifactorial disease [11]. One of the contributing factors is the suppressive effect of BPs on various bone cells [31,32]. The most common type of BPs are nitrogen-containing BPs (N-BPs), which inhibit farnesyl diphosphate synthase in the mevalonate pathway (MVP) [33]. Several enzymes within the MVP are potential targets of N-BPs and inhibiting them leads to the loss of prenylation of small GTP-binding proteins, including Ras, Rho, Rab, Arf, and Ran [34,35,36]. Combined with their high affinity for mineral tissue [37,38], N-BPs exhibit a long-lasting detrimental effect on the bone environment [39]. Suppressive effects of BPs are not limited to osteoclast-mediated bone resorption. They also reduce bone formation by osteoblasts, thereby leading to impaired bone remodeling. This mechanism may potentially contribute to MRONJ [31,40].

Numerous studies have investigated the suppressive effects of BPs on osteoblasts, particularly on osteogenesis [8,13,14,15,16]. These studies indicate that BPs inhibit osteoblast viability and proliferation, induce apoptosis, and downregulate the expression of proteins involved in osteoblast differentiation, leading to decreased mineralization [8,13,14,15,16]. Our findings align with those of previous studies [8,9,13,14,15,16,17,18], demonstrating that Zol suppressed cell viability, induced apoptosis, inhibits cell migration, and downregulated the gene and protein expression associated with cell differentiation and bone mineralization.

EMD refers to the purified extract of the naturally developing enamel matrix proteins that are present during the secretory stage of dental crown development [25]. The application of EMD stimulates local growth factor expression, extracellular matrix deposition, mineral deposition, and wound healing, similar to the early processes of tooth development and alveolar bone formation [41,42]. Therefore, EMD is considered to have regenerative potential, supporting bone formation as an osteo-promotive agent [27]. EMD alone is not osteogenic, meaning it does not produce ectopic bone formation; however, it was shown to have an osteo-promotive effect on bone regeneration, especially when combined with bone graft materials [43]. Furthermore, EMD induces the proliferation of microvascular endothelial cells and promotes angiogenesis, which is crucial for bone tissue wound healing [44].

Although the exact cellular mechanisms by which EMD influences osteoblast behavior remain unclear, several studies have indicated that EMD can stimulate the secretion and activation of growth factors such as TGF-β and BMP [27,45,46,47]. TGF-β plays a critical role in bone remodeling, including matrix protein synthesis, and directly affects osteoblasts through processes such as proliferation or differentiation, depending on their osteoblastic cell phenotypes or differentiation stages [48]. Schwartz et al. showed that the effect of EMD on osteoblasts varies depending on the maturation stage of the osteoblastic lineage [49]. Other studies have demonstrated stage-specific effects of EMD on osteoblast differentiation, suggesting that EMD may be particularly effective in driving osteoblast differentiation in less mature or more undifferentiated cells [50,51].

This study demonstrated an elevated apoptosis rate in hFOB and MG63 cells following treatment with Zol, which was reversed by the addition of EMD. Tumor necrosis factor alpha (TNF-α), an inflammatory cytokine, mediates apoptosis in response to infection or injury [52], and a study demonstrated that EMD could reduce TNF-α-induced apoptosis similarly to the effects seen with TGF-β [53]. Therefore, EMD may stimulate proliferation and prevent apoptosis of osteoblasts under the influence of BP, thereby enhancing bone remodeling.

Our results suggest that EMD has a stimulatory effect on cell migration, which mitigates the negative effects of BPs. Although one study indicated that EMD has a minimal effect on cell migration [54], other studies suggested that EMD promoted the migration of human umbilical vein endothelial cells in both the wound-healing and Boyden chamber assays [44,55]. The exact mechanism underlying this increased migration remains unclear; however, it has been hypothesized that EMD accelerates cell migration through chemotaxis [55,56].

Several experimental studies have also demonstrated that EMD enhances the expression of genes associated with osteoblast differentiation and mineralization [49,57,58,59,60,61,62]. EMD influences osteoblast differentiation across various cell models, including MG-63, MC3T3-E1, Kusa/A 1, and 2T9 cells [49,57,58,59,60,61], and it promotes osteogenesis by upregulating bone-associated genes such as ALP, OCN, and BSP, thereby stimulating mineral nodule formation [62]. In our study, the decreased gene expression of ALP due to BPs was reversed by the co-addition of EMD in both of the cell lines examined. This trend was also observed for OC and RANKL expression in the hFOB cells. Although the suppressive effects on M-CSF, BSP, and COL1 genes were not profound, the addition of EMD further increased their expression. However, there were discrepancies in the responses of several genes across different cell lines. Several factors can contribute to these variations, including differences in cell line characteristics, the stage of differentiation, and heterogeneity within each cell line. Different cell lines exhibit unique behaviors and characteristics owing to genetic variations, mutations, or adaptations that occur during isolation and culture [63]. Consequently, their responses to external stimuli, such as drug treatments, can vary significantly. There was also a trend indicating that BP treatment suppressed protein expressions, and the addition of EMD to the Zol treatment facilitated the recovery of the expression levels of several measured proteins. However, the gene expression levels were not entirely consistent. Considering that BPs inhibit the prenylation of proteins [34,35,36], it is possible that gene expression does not always correlate with protein expression during Zol treatment.

EMD is widely used in periodontal therapy and has demonstrated effectiveness in tissue regeneration [64,65,66]. Building on previously proven effects, this study suggests that EMD mitigates the effects of Zol, resulting in the recovery of cell survival, migration, and gene and protein expression. However, the behavior of the osteoblasts was not fully restored, and further studies are necessary to confirm their effects at the cellular level. Moreover, the exact mechanism of action of EMD remains unknown, and results may vary in in vivo conditions. Different EMD concentrations may lead to better results, suggesting that optimizing the dosage could enhance its therapeutic effectiveness. Nevertheless, EMD does not require general administration, and no negative effects on bone physiology have been reported. Therefore, if dentoalveolar surgery such as tooth extraction, periodontal, or implant surgery is planned for patients with a history of BP therapy or those currently undergoing such therapy, the concomitant use of EMD during the surgical procedure may reduce the risk of MRONJ development or implant failure. Additionally, it can be applied in the surgical treatment of MRONJ with the expectation of reducing the recurrence of osteonecrosis. Pentoxifylline or teriparatide have been reported as preventive and adjuvant treatment strategies for MRONJ through pharmacological approaches [67,68]. However, these treatments require systemic administration, whereas EMD can be applied locally to adjacent teeth and bony surfaces during surgery. EMD is already commercially available, indicating its potential for broader clinical applications. However, further preclinical studies and clinical trials are essential to confirm the efficacy and safety of EMD and to develop an optimal strategy for its application.

## Figures and Tables

**Table 1:** PCR primers designed for genes encoding alkaline phosphatase (ALP), osteocalcin (OC), receptor activator of nuclear factor kappa-B ligand (RANKL), macrophage colony stimulating factor (M-CSF), bone sialoprotein (BSP), type 1 collagen (COL1).

Gene		5′→3′
ALP	F	GACAAGAAGCCCTTCACTGC
	R	AGACTGCGCCTGGTAGTTGT
OC	F	GGCGCTACCTGTATCAATGG
	R	TCAGCCAACTCGTCACAGTC
RANKL	F	CACTATTAATGCCACCGAC
	R	GGGTATGAGAACTTGGGATT
M-CSF	F	TAGCCACATGATTGGGAGTG
	R	TATCTCTGAAGCGCATGGTG
BSP	F	GCGAAGCAGAAGTGGATGAAA
	R	TGCCTCTGTGCTGTTGGTACTG
COL1	F	CCTGGTGCTAAAGGAGAAAGAGG
	R	ATCACCACGACTTCCAGCAGGA
GAPDH	F	AACAGCGACACCCACTCCTC
	R	CATACCAGGAAATGAGCTTGACAA

## Data Availability

The raw data supporting the conclusions of this article will be made available by the authors upon request.
